# EHMTI-0088. Prediction of treatment response in chronic daily headache patients (a prospective study)

**DOI:** 10.1186/1129-2377-15-S1-D23

**Published:** 2014-09-18

**Authors:** V Golovacheva, V Parfenov, G Tabeeva

**Affiliations:** 1 Neurology, I.M.Sechenov The First Moscow State Medical University, Moscow, Russia

## Introduction

The prevalence of chronic daily headache (CDH) is 4-5% in general population. In headache clinics about 40% patients have CDH and 50-82% of them overuse acute drugs. Combining pharmacological and behavioural therapies can increase effectiveness of treatment, but predictive factors of treatment response are still discussed.

## Aims

To examine predictive factors of treatment response for patient with CDH

## Methods

35 patients with CDH in age from 23 to 78 years were included in the study. All patients were treated by pharmacological therapy and cognitive-behavioural therapy. Patients with medication-overuse headache (MOH) underwent withdrawal therapy. Prospective analysis of clinical-psychological characteristics was performed at the end of 1 and 3 months of treatment.

## Results

40% (N=14) of patients were classified as having chronic migraine (CM), 25,7% (N=9) had chronic tension-type headache (CTTH), 31,4% (N=11) had CTTH and episodic migraine, 2,9% (N=1) had hemicrania continua. 62,9% (N=22) patients suffered from comorbid psychiatric conditions. Of those, 15,6% (N=5) had mood disorder, 17,1% (N=6) - anxiety disorder,14,3% (N=5) - personality disorder and 17,1% (N=6) suffered from somatization disorder. 60% (N=21) of participants experienced at least 50% reduction of headache frequency after 3 months of treatment. Predictive factors of poor treatment response were: 1) certain psychiatric disorders (mood disorder, somatization disorder, schizoid personality disorder); 2) duration of CDH is more than 5 years; 3) out of employment.

## Conclusion

Predictive factors of poor treatment response are mood disorder, somatization disorder, schizoid personality disorder, more than 5-years duration of CDH and out of employment.

No conflict of interest.

